# P2Y_12_ Receptor on the Verge of a Neuroinflammatory Breakdown

**DOI:** 10.1155/2014/975849

**Published:** 2014-08-07

**Authors:** Susanna Amadio, Chiara Parisi, Cinzia Montilli, Alberto Savio Carrubba, Savina Apolloni, Cinzia Volonté

**Affiliations:** ^1^Santa Lucia Foundation, Via del Fosso di Fiorano 65, 00143 Rome, Italy; ^2^Cellular Biology and Neurobiology Institute (CNR), Via del Fosso di Fiorano 65, 00143 Rome, Italy

## Abstract

In the CNS, neuroinflammation occurring during pathologies as amyotrophic lateral sclerosis (ALS) and multiple sclerosis (MS) is the consequence of an intricate interplay orchestrated by various cell phenotypes. Among the molecular cues having a role in this process, extracellular nucleotides are responsible for intercellular communication and propagation of inflammatory stimuli. This occurs by binding to several receptor subtypes, defined P2X/P2Y, which are widespread in different tissues and simultaneously localized on multiple cells. For instance, the metabotropic P2Y_12_ subtype is found in the CNS on microglia, affecting activation and chemotaxis, on oligodendrocytes, possessing a hypothesized role in myelination, and on astrocytes. By comparative analysis, we have established here that P2Y_12_ receptor immunolabelled by antibodies against C-terminus or second intracellular loop, is, respectively, distributed and modulated under neuroinflammatory conditions on ramified microglia or myelinated fibers, in primary organotypic cerebellar cultures, tissue slices from rat striatum and cerebellum, spinal cord sections from symptomatic/end stage SOD1-G93A ALS mice, and finally autoptic cortical tissue from progressive MS donors. We suggest that modulation of P2Y_12_ expression might play a dual role as analytic marker of branched/surveillant microglia and demyelinating lesions, thus potentially acquiring a predictive value under neuroinflammatory conditions as those found in ALS and MS.

## 1. Introduction

A basic set of proteins and mRNAs are differentially expressed among cell types, temporally and spatially, generating a vast assortment of cell phenotypes and/or activation states within a single tissue. Outlining this protein/mRNA portrait is thus crucial for understanding not only the uniqueness characterizing cells, but especially their distinguished functions [1]. This becomes of major relevance when the balance between cell-intrinsic properties and identity cues received and provided by each cell to its neighboring cells then shapes the cell-to-cell cross talk during physiopathological conditions. In the CNS, neuroinflammation is the typical consequence of the interchange among different cell types, particularly neurons, astrocytes, oligodendrocytes and microglia, of a variety of cues as neurotransmitters, cytokines, chemokines, toxic metabolites that condition the final protein/mRNA profiles of cells, their activation states and functional outcomes [2, 3]. Since neuroinflammation accompanies a large variety of neurodegenerative diseases, there is increasing interest in determining how the different cell phenotypes and cellular interconnectivity might contribute to reduce inflammation and reverse neurodegeneration.

Microglia actively participate to the context-dependent, neuroprotective/neurotoxic molecular network that is triggered during neuroinflammation [4]. Among the molecular cues having a key role in this process, extracellular nucleotides are major responsible for intercellular communication and propagation of inflammatory stimuli [5–7]. This occurs by specific binding to various receptor subtypes, termed ionotropic P2X and metabotropic P2Y, which are simultaneously localized on several different cell phenotypes. Among these, the P2Y_12_ receptor subtype [8] belonging to the Gi class of G protein-coupled receptors is activated by ADP. Two transcript variants apparently encoding the same protein isoform have been identified so far for P2RY_12_ gene [8], but the determinants for cell specificity of P2Y_12_ protein expression are still unknown.

P2Y_12_ is found on the surface mainly, but not exclusively, of blood platelets, where it acts as blood clotting regulator and target for the treatment of thromboembolisms [9, 10]. In the nervous system, the tissue- and cellular-selective expression of P2Y_12_ exhibits a pattern throughout white and gray matter consistent with astrocyte expression [8], although it has not been found colocalization between P2Y_12_ and GFAP-positive astrocytes in rat brain cortex and nucleus accumbens, despite the abundant presence of P2Y_12_ mRNA [11]. Moreover, we previously established* in vivo* the expression of P2Y_12_ in oligodendrocytes and myelin sheaths of rat cerebral cortex, subcortical areas, and periventricular white matter. This localization is confirmed throughout the corticospinal tract, therefore suggesting high conserved tissue-homogeneity and phenotype-specificity, and a hypothesized role in myelination [12–15]. P2Y_12_ is finally observed in brain and spinal cord resident microglia, where it affects activation, chemotaxis [16–19] and neuropathic pain [20], but it is not observed, for example, in peripheral macrophages in spleen [18, 20]. P2Y_12_ expression in primary microglia is variable with postnatal development and shows sexually dimorphic behavior [21].

Through the use of all the available P2Y_12_-immunoreactive antibodies recognizing the C-terminus or the second intracellular loop of the receptor, the aim of the present work is to provide comparative evidence about P2Y_12_ cell specificity in microglia versus oligodendrocytes particularly from the healthy and diseased CNS under neuroinflammatory conditions as those sustained during amyotrophic lateral sclerosis (ALS) and multiple sclerosis (MS).

## 2. Materials and Methods

### 2.1. Animals

Adult B6.Cg-Tg (SOD1-G93A)1Gur/J mice expressing high copy number of mutant human SOD1 with a Gly93Ala substitution (SOD1-G93A) were originally obtained from Jackson Laboratories (Bar Harbor, ME, USA) and housed in our indoor animal facility as described in Apolloni and collaborators, [22]. The animals were euthanized, according to the guidelines for preclinical testing and colony management [23]. Also neonatal Wistar and adult Lewis rats (from Charles River Laboratories, LC, Italy) were housed in our indoor animal facility.

Animal procedures were performed according to European Guidelines for the use of animals in research (86/609/CEE) and the requirements of Italian laws (D.L. 116/92). Ethical procedures were approved by Animal Welfare Office, Department of Public Health and Veterinary, Nutrition and Food Safety, General Management of Animal Care and Veterinary Drugs of the Italian Ministry of Health. All efforts were made to minimize animal suffering and number of animals necessary to produce reliable results.

### 2.2. Mouse Microglia Primary Cultures

Microglia primary cultures prepared from mouse cortex as previously described [24] were about 98% pure, as verified by immunofluorescence with CD11b (for microglia), glial fibrillary acidic protein (GFAP, for astrocytes), neuronal nuclei (NeuN, for neurons), and SMI94 (for oligodendrocytes).

### 2.3. Rat Microglia and Oligodendrocyte Primary Cultures

Purified cultures of oligodendrocytes were prepared from forebrain of postnatal day 1-2 Wistar rats, according to minor modifications from a previously described method [25]. After removing the meninges, cortices were minced, digested, and dissociated by passage through 70 *μ*m nylon cell strainer (BD Biosciences, Europe). Cells were plated in Dulbecco's modified Eagle's medium (DMEM) supplemented with 20% heat-inactivated fetal bovine serum (FBS), 4 mM glutamine, 1 mM sodium pyruvate, 50 U/mL penicillin, 50 *μ*g/mL streptomycin, and 100 *μ*g/mL gentamicin in T75 poly-D-lysine-coated flasks, at about 10 million cells/flask. The cultures were maintained at 37°C in a 5% CO_2_ and 95% air atmosphere for 14 days. Mixed glial cultures were then shaken at 200 rpm at 37°C for 1 h and 98% pure microglia collected from the supernatant of each flask, as verified by immunofluorescence with GFAP, NeuN, Neural/Glial antigen 2 (NG2, for oligodendroglial precursor cells), myelin basic protein (MBP, for mature oligodendrocytes), and CD11b. After further shaking at 200 rpm and 37°C for 15–18 h, the detached cell suspension was finally incubated for 1 h at 37°C for differential adhesion of contaminating cells. The non-adherent cells were filtered through 40 *μ*m nylon cell strainer (BD Biosciences), spun for 10 min at 100 g, resuspended in oligodendroglial precursor cells medium (basal chemically defined medium: DMEM, 4 mM L-glutamine, 1 mM sodium pyruvate, 0.1% bovine serum albumin (BSA), 50 *μ*g mL^−1^ apo-transferrin, 5 *μ*g mL^−1^ insulin, 30 nM sodium selenite, 10 nM D-biotin, and 10 nM hydrocortisone) containing 10 ng mL^−1^ platelet derived growth factor-AA (PDGF-AA) and 10 ng mL^−1^ basic fibroblast growth factor (bFGF), and seeded at the density of 1 × 10^4^ cells/cm^2^ into poly D,L-ornithine-coated plates. The cells were then induced to differentiate into mature oligodendrocytes when the basal chemically defined medium was added with 15 nM triiodothyronine, 10 ng mL^−1^ ciliary neurotrophic factor (CNTF) and 0,05 mg 10 mL^−1^ N-acetyl-L-cysteine (NAC), for 4–7 days. A 98% pure population of oligodendrocytes was thus obtained, as verified by immunofluorescence with NG2, MBP, GFAP, NeuN, and CD11b antibodies.

### 2.4. Cerebellar Organotypic Cultures

Organotypic cerebellar slice cultures were prepared with modifications from previously published protocols [26, 27]. Briefly, cerebella were excised from neonatal Wistar rat (8–10 days old), cut on a Mc Ilwain tissue chopper (400 *μ*m) and parasagittal slices separated into cold Hank's balanced salt solution (HBSS). Two to three slices were plated on each Millicell CM culture inserts (Millipore, Italy) and kept in organotypic maintenance medium (50% Basal Medium Eagle-BME, 25% HBSS, 25% heat-inactivated horse serum, supplemented with 5 mg/mL glucose, 1 mM glutamine, 50 U/mL penicillin, and 50 *μ*g/mL streptomycin) at 37°C in a 5% CO_2_ and 95% air atmosphere. The medium was changed every 2 days and double immunofluorescence was performed in free floating after 7–10 days* in vitro*. Organotypic cultures were washed three times with PBS, fixed with 4% paraformaldehyde for 1 h, rinsed, and blocked for 1 h in Phosphate buffered saline (PBS)/10% normal donkey serum (NDS)/0.4% Triton X-100. Primary antibodies were incubated for 24 h in PBS/2% NDS/0.4% Triton X-100 (Table 1). The secondary antibodies used for double labelling are Cy3-conjugated donkey anti-rabbit IgG (1 : 100, Jackson Immunoresearch, Europe) or Alexa Fluor 488-AffiniPure donkey anti-mouse IgG (1 : 200, Jackson Immunoresearch). After rinsing, sections were mounted, covered with Fluoromount medium (Sigma-Aldrich, Milan, Italy) and a coverslip, and analyzed by confocal microscopy.

### 2.5. Immunofluorescence on Mouse Microglia and Rat Oligodendrocytes

Microglia and oligodendrocytes were washed three times with PBS, fixed with 4% paraformaldehyde for 10 min (oligodendrocytes) or 20 min (microglia), washed, permeabilized with 0.05–0.1% Triton X-100 for 10 min, rinsed, and blocked for 30 min in PBS/1% BSA. Microglia were stained for about 3 h at 37°C in 1% PBS/BSA with 5 *μ*g/mL Cy2-phalloidin (Sigma-Aldrich), in combination with primary antibodies against P2Y_12_ receptor, as reported in Table 1. Oligodendrocytes were stained with primary antibodies against P2Y_12_ receptor and MBP or NG2. The secondary antibodies used for double labelling are Cy3-conjugated donkey anti-rabbit IgG (1 : 100, Jackson Immunoresearch) or Alexa Fluor 488-AffiniPure donkey anti-mouse IgG (1 : 200, Jackson Immunoresearch). Cells were extensively washed and stained with the nucleic acid blue dye, Hoechst 33342 (1 : 1000). After rinsing, cells were covered with Fluoromount medium (Sigma-Aldrich) and a coverslip and analyzed by confocal microscopy.

### 2.6. Histological Procedures and Immunofluorescence on Mouse and Rat Tissue

Animals were anaesthetized by intraperitoneal injection of chloral hydrate (500 mg/Kg) and transcardially perfused with PBS followed by 4% paraformaldehyde at pH 7.4. Tissue samples (mice spinal cord and rat brain) were then postfixed for 1-2 days, and cryoprotected in 30% sucrose in PBS at 4°C. Tissues were stored at −80°C.

Mice spinal cords (L3–L5) were cut at 30 *μ*m thickness with a frozen microtome. Sections were mounted on slide glasses and allowed to air-dry (1-2 h). A rectangle was then drawn around the sections with a PAP pen (Sigma-Aldrich). Rat brains were cut at 40 *μ*m thickness using a cryostat microtome and sections were processed in free-floating. Double immunofluorescence analysis was performed after blocking in PBS containing 10% NDS and 0.3% Triton X-100 for 1 h at room temperature. Sections were incubated with different combinations of primary antibodies (Table 1), in PBS, 0.3% Triton X-100 and 2% NDS, for 24–48 h at 4°C. Finally, sections were washed with PBS and incubated with appropriate fluorescent-conjugated secondary antibodies for 3 h at room temperature. After rinsing, sections were covered with Fluoromount medium (Sigma-Aldrich) and a coverslip and analyzed by confocal microscopy.

### 2.7. Human Tissue Source, Lesion Detection, Classification and Immunofluorescence

Tissues supplied by UK Multiple Sclerosis Tissue Bank at Imperial College, London, were collected postmortem with fully informed consent from both donors and close relatives. Procedures for retrieval, processing, and storage have gained ethical approval from all appropriate committees. The brain tissues analyzed were from 7 neuropathologically confirmed cases of secondary progressive MS (SPMS). Analysis was performed also on samples from patients who died due to nonneurological diseases (healthy). Cerebral hemispheres were fixed with 4% paraformaldehyde for about two weeks, coronally sliced, and blocked. Individual blocks were cryoprotected in 30% sucrose for one week and frozen by immersion in isopentane precooled on a bed of dry ice. Frozen tissue blocks were stored at −80°C. Cryostat sections (30–40 *μ*m thick) were either stained with Luxol fast blue and cresyl fast violet (Kluver-Barrera staining), in order to detect white matter lesions and their cellularity, or subjected to immunohistochemistry for MBP, in order to distinguish grey matter lesions. Sections were processed in free-floating for double immunofluorescence studies, as described in Amadio and collaborators [13].

### 2.8. Confocal Analysis

Immunofluorescence was analyzed by means of a confocal laser scanning microscope (Zeiss, LSM700, Germany) equipped with four laser lines: 405, 488, 561, and 639 nm. The brightness and contrast of the digital images were adjusted using the Zen software (Zeiss).

### 2.9. Plasmid Construction and Transfection

Human P2Y_12_ complete cDNA was obtained by reverse transcription with enhanced avian RT-PCR kit (Sigma-Aldrich) from total human brain RNA (Life Technologies, Paisley, UK). The obtained cDNA was then cloned into the XhoI and XbaI sites of the eukaryotic expression vector CS2 + MT to provide N-terminal 6X c-Myc epitope-tagged mammalian expression plasmids, which has been validated by DNA sequencing. Oligos used for amplification were as follows:* forward* 5′gcCTCGAGatgcaagccgtcgacaacctc3′ and reverse 5′gcTCTAGAgtttacattggagtctcttc designed on human P2Y_12_ mRNA (NM_022788.3).

Human embryonic kidney 293 cells (HEK 293) and Sloan-Kettering neuroblastoma SH-SY5Y clone (SH-SY5Y) cells were grown in DMEM supplemented with 10% FBS, 100 unit/mL penicillin, and 100 *μ*g/mL streptomycin at 37°C in atmosphere containing 5% CO_2_. One day before transfection, HEK293 or SH-SY5Y cells were plated and transfection of P2Y_12_-CS2 + MT or CS2 + MT empty vector was performed with lipofectamine 2000 (Life Technologies), according to manufacturer instructions.

### 2.10. RT-PCR

Primary rat microglial and oligodendrocyte cells were lysed with TRIzol (Life Technologies) and total RNA was extracted following the manufacturer's instructions. After DNase treatment (Qiagen, Hilden, Germany), equal amount of total RNA (1 *μ*g) was subjected to retrotranscription by high capacity RNA-to-cDNA kit (Life Technologies) and 50 ng of each cDNA were amplified with rat P2Y_12_ specific primers (F: 5′-GATTGATAACCATTGACC-3′; R: 5′-GGTGAGAATCATGTTAGG-3′). The number of cycles was fixed to 35. Amplification products (10 *μ*L of 20) were electrophoresed on 2% agarose gel containing ethidium bromide (1 *μ*g/mL, Sigma Aldrich), photographed under UV light using Kodak Image Station 440CF, with Molecular Imaging Software 4.0.1.

### 2.11. Protein Extraction, SDS-PAGE and Western Blotting

In order to isolate total protein extracts, microglia and oligodendrocytes were harvested with ice-cold RIPA buffer (PBS, 1% Nonidet P-40, 0.5% sodium deoxycholate, and 0.1% SDS), added with protease inhibitor cocktail (Sigma Aldrich). Lysates were kept for 30 min on ice and then centrifuged for 10 min at 14,000 rpm, at 4°C. Snap-frozen blocks from cases MS114, MS125, and MS163 supplied by UK Multiple Sclerosis Tissue Bank at Imperial College in London were homogenized in RIPA buffer, added with protease inhibitor cocktail, incubated on ice for 1 h, and centrifuged at 14,000 rpm for 10 min at 4°C. Rat brain and mouse brain and spinal cord were homogenized and sonicated in RIPA buffer, added with protease inhibitor cocktail, kept for 1 h on ice, and centrifuged at 4°C for 10 min at 13,000 rpm. Supernatants were collected and analyzed for protein content by Bradford colorimetric assay (Biorad, Milan, Italy). Analysis of protein components was performed by polyacrylamide gel (SDS-PAGE) separation (BioRad) and transfer onto nitrocellulose membranes (Amersham Biosciences, Cologno Monzese, IT). After saturation, blots were probed overnight at 4°C, with the specified antibody (Table 1), and finally incubated for 1 h with HRP-conjugated secondary antibodies and detected on X-ray film (Aurogene, Rome, Italy), using ECL Advance Western blotting detection kit (Amersham Biosciences). Quantifications were performed by Kodak Image Station. P2Y_12_ protein was detected with molecular mass comprised between 40 kDa (as predicted by amino acid sequence) and 49-50 kDa (as predicted by the manufacturer data sheet).

### 2.12. Statistical Analysis

Data are presented as mean ± standard error of the mean (SEM). Analysis was performed with the statistical software package MedCalc (Medcalc Software, Mariakerke, Belgium). Statistical differences between groups were verified by Student's *t*-test. *P < 0.05 was considered significant.

## 3. Results

In order to provide wide ranging comparative analysis of P2Y_12_ expression particularly in microglia and oligodendrocytes under neuroinflammatory conditions, we performed immunofluorescence and confocal analysis of receptor expression in primary cortical and organotypic cerebellar cultures, in tissue slices from rat striatum and cerebellum, in spinal cord sections from symptomatic and end stage SOD1-G93A ALS mouse model, finally in autoptic cortical tissue from progressive MS donors.

### 3.1. Mapping the Recognition Sites of P2Y_12_ Antibodies on Receptor Protein and Antibodies Validation

**Figure 1:**
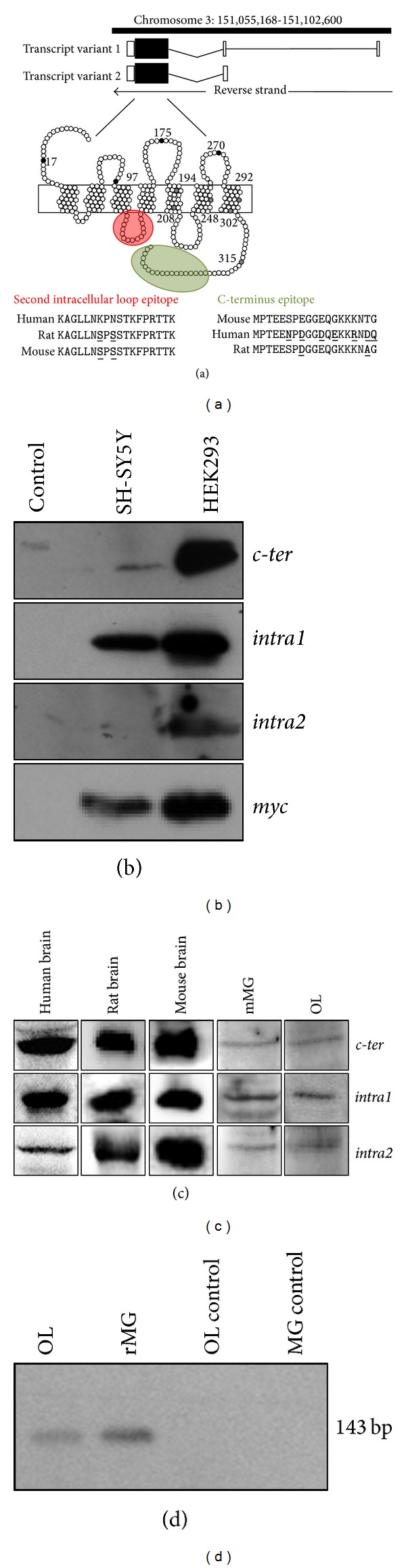
P2Y_12_ antibodies validation and RT-PCR analysis. (a) Scheme of human P2RY_12_ gene location, transcript variants [29, 30], and protein structure, with amino acid epitopes recognized by the used antibodies (Table 1) and highlighted in color (*intra1*,* intra2*, and* intra fl*, red circle;* c-ter*, green oval). Species conservation for each epitope was calculated by using BLAT tool of UCSC genome browser [31]. (b) Total protein extracts from SH-SY5Y or HEK293 cells expressing Myc-tagged P2Y_12_ receptor were subjected to Western blot analysis with the indicated antibodies. (c) Total protein extracted from human, rat and mouse brain, from primary mouse microglia (mMG) and rat oligodendrocyte (OL) cultures were subjected to Western blot analysis with the indicated antibodies. For* intra2* antibodies,  lots AN01/02/04/0502/0602 were used. (d) RT-PCR using primers specific for P2Y_12_ mRNA was performed on total RNA from rat microglia (rMG) and OL. Control lanes show RT-PCR performed without reverse transcriptase enzyme.

P2Y_12_ receptor is formed by two transcript variants that give rise to identical proteins with 342 amino acids, a secondary structure constituted by seven hydrophobic transmembrane domains connected by three extracellular and three intracellular loops, with four extracellular cysteine residues most likely contributing to the nucleotide binding site [28] (Figure 1(a)). The commercially available P2Y_12_ antibodies that we mostly used in our work are raised against the second intracellular loop, and precisely amino acids 125–142 (here named* intra1*,* intra2*, and* intra fl*; see red circle in Figure 1(a)), and against the C-terminus, here named* c-ter* (see green oval in Figure 1(a)) (see also Table 1).

In order to validate the use of these different antibodies for P2Y_12_ receptor, we compared them on recombinant P2Y_12_ receptor protein obtained by cloning the complete cDNA of the human receptor into the eukaryotic expression vector CS2 + MT, to provide the expression of a N-terminal c-Myc tagged fusion protein. After transfection into SH-SY5Y and HEK293 cell lines, we analyzed total protein extracts by SDS-PAGE and Western blotting using* c-ter*,* intra1*, and* intra2* antibodies. Although with different intensities, all the antibodies recognize the myc-P2Y_12_ protein band at the predicted molecular mass of 50 kDa. No signal is detected when transfection is performed with empty vector (control). These results confirm the specificity of the used antibodies toward denatured recombinant P2Y_12_ receptor (Figure 1(b)).

In order to verify the expression of P2Y_12_ mRNA in rat primary oligodendrocytes (OL) and microglia (rMG), RT-PCR was performed using specific primers designed on receptor sequence. As shown in Figure 1(d), RT-PCR on both rMG and OL reveals the presence of the predicted P2Y_12_ mRNA band. The absence of DNA contamination is confirmed in empty control lanes.

Next, we validated the antibodies with protein extracts from dissociated primary cultures or tissues from different species. P2Y_12_ protein is specifically recognized in extracts from human cerebral cortex snap frozen tissue, rat and mouse brain, mouse primary microglia (mMG) and OL (Figure 1(c)). No signals are obtained when the immunoreactions are performed in the presence of P2Y_12_ neutralizing peptides, when available from manufacturer (data not shown).

### 3.2. Presence of P2Y_12_ Receptor in Dissociated and Organotypic Primary Cultures

**Figure 2:**
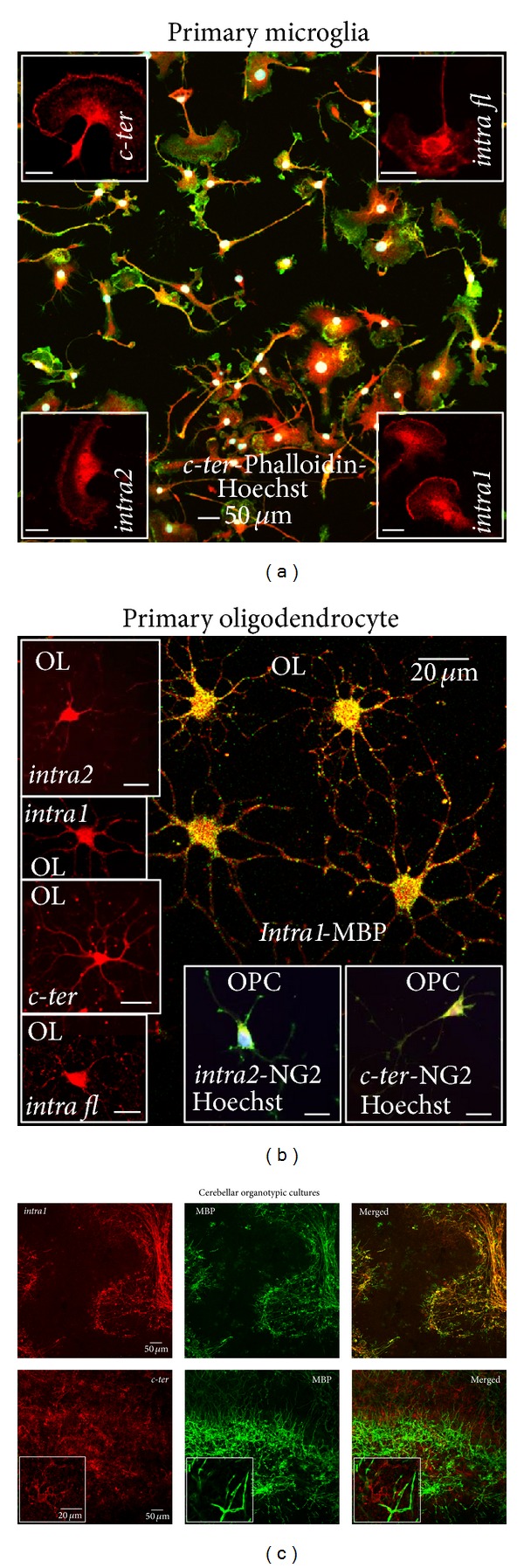
P2Y_12_ receptor in dissociated and organotypic primary cultures. (a) Mouse primary cortical microglia were subjected to immunofluorescence and confocal analysis with phalloidin (green, merged field) and P2Y_12_ receptor antibodies (red, insets and merged) and Hoechst (white, merged). Scale bars in insets: 20 *μ*m. (b) Double immunofluorescence and confocal analysis of primary rat mature (OL) and precursor (OPC) oligodendrocytes was performed with antibodies for P2Y_12_ receptor, MBP, NG2 (Table 1). For* intra2* antibodies, lots AN01/02/04/0502/0602 were used. (c) Rat cerebellar organotypic cultures were analyzed by double immunofluorescence and confocal microscopy for* intra1* (red) and* c-ter *(red), highlighting different structures (see also insets), and MBP (green).

In order to establish cell specificity of P2Y_12_ receptor expression, we performed comparative immunofluorescence and confocal analysis in primary dissociated cortical microglia and oligodendrocytes (Figures 2(a) and 2(b)), as well as organotypic cerebellar cultures (Figure 2(c)). P2Y_12_ is strongly recognized by both C-terminus- and second intracellular loop-recognizing antibodies (red,* c-ter*, upper left inset;* intra fl*, upper right inset;* intra1*, lower right inset and* intra2*, lower left inset), specifically distinguishing the very heterogeneous morphological features of mouse primary microglia, as shown by double fluorescence and confocal analysis performed with phalloidin (green), a marker for filamentous actin (Figure 2(a)). Fan-like cells (insets), elongated rod-like cell bodies with short and tiny branches, asymmetrical hairy cells with miniature processes or lamellipodia are simultaneously observed (*c-ter* Phalloidin Hoechst, merged). Likewise, all the P2Y_12_ antibodies (*intra2*,* intra1*,* c-ter*, and* intra fl*) recognize the multibranched morphology of rat mature oligodendrocytes (Figure 2(b), OL, insets, red) in primary cultures, as confirmed by double immunofluorescence and confocal analysis with MBP antibody (OL,* intra1*-MBP, yellow merged image). In addition, both* intra2* and* c-ter* antibodies distinguish the NG2-positive rat oligodendrocyte precursor cells (OPC, merged insets).

P2Y_12_ immunoreactivity is confirmed in the* ex-vivo* system of organotypic rat cerebellar cultures (Figure 2(c)). However, differently from mouse microglia and rat oligodendrocyte primary cultures, the second intracellular loop- (red,* intra1*) and C-terminus-recognizing (red,* c-ter*) antibodies surprisingly immunoreact with different cell phenotypes when the integrity and cytoarchitecture of the tissue is preserved, as in organotypic cultures. While* intra1* antibody (red) exclusively labels MBP-positive fibers (green) and highlights myelin structures,* c-ter* antibody does not immunoreact with myelinated fibers, being present on cells likely resembling microglia (insets), as also confirmed by colocalization with microglial marker CD11b (data not shown). Results similar to those found with* intra1* were also obtained with* intra2* antibodies, lots AN01/02/04 (data not shown).

### 3.3. Cell-Selective Presence of P2Y_12_ Receptor in Rat Brain Tissue

**Figure 3:**
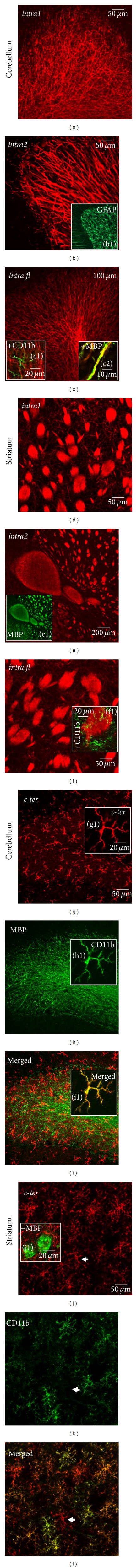
P2Y_12_ receptor in rat brain tissue. Double immunofluorescence and confocal analysis was performed on sections from rat cerebellum (panels (a), (b), (c), (g), (h), and (i)) and striatum (panels (d), (e), (f), (j), (k), and (l)) with* intra1*,* intra2*-lots AN01/02/04,* intra fl*,* c-ter* (all red), and GFAP (green, inset b1), CD11b (green, insets c1, f1, h1; yellow merged, inset i1; green, panel (k); merged, panel (l)), MBP (yellow merged, inset c2; green, inset e1; green, panels (h) and (i); green, inset j1) antibodies.

We next verified if the cell type-specific presence of P2Y_12_ receptor observed in organotypic culture, either in myelin structures or in microglia, respectively,  by the use of the second intracellular loop-(*intra1*) or the C-terminus-recognizing (*c-ter*) antibody, is also confirmed in rat cerebellar and striatal slice tissues (Figure 3). All the antibodies raised against the second intracellular loop (red,* intra1*,* intra2*,* intra fl*) identify the abundant presence of P2Y_12_ receptor only on myelinated fibers from both cerebellum (Figures 3(a), 3(b), and 3(c)) and striatum (Figures 3(d), 3(e), and 3(f)), as confirmed by colocalization of signals obtained with* intra fl* and MBP antibodies in cerebellum (+MBP, inset c2, merged, yellow) and striatum (data not shown); colocalization of signals obtained with* intra1* and* intra2* with MBP antibodies in cerebellum and striatum (data not shown); immunoreactivity of* intra1*,* intra2*, and* intra fl* antibodies for structures identical to those observed in cerebellum (data not shown) and striatum with MBP antibody (see for instance MBP, inset e1, green); identification by second intracellular loop P2Y_12_ antibodies of structures totally different from both Bergmann glia and astrocytes recognized by specific GFAP antibody in cerebellum (inset b1, green), and from microglia identified by specific CD11b antibody in cerebellum (+CD11b, inset c1, merged, green) and striatum (+CD11b, inset f1, merged, green). Of notice, all the 2nd intracellular loop antibodies are able to describe the specific cytoarchitecture of both cerebellum, where radiant and sparse fibers clearly characterize the* lobuli*, and of striatum, where white matter is instead organized in distinct bundles.

As in organotypic cultures, the C-terminus antibody (red,* c-ter*) instead recognizes only microglia in rat cerebellum (Figures 3(g), 3(h), and 3(i), and insets g1, h1, i1), striatum (Figures 3(j), 3(k), and 3(l)) and cerebral cortex slices (data not shown), with a signal more uniformly distributed throughout the whole tissue. By comparing the CD11b and* c-ter* immunolabelling in the striatum, we furthermore observe that the mutual intensity of signals is cell-selective within the microglia population, with some cells exclusively positive for* c-ter* (arrow), and others instead showing different grades of CD11b-*c-ter* colocalization (see for instance orange, yellow and greenish cells in the merged field). Regrettably, double immunofluorescence with* c-ter* and Iba1 microglia marker [32] is not practicable, since both antibodies are raised in the same species. However, no colocalization of signals is ever shown between MBP (green) and* c-ter* antibodies (+MBP, inset j1, merged).

### 3.4. P2Y_12_ Expression Is Modulated during Neuroinflammation in Spinal Cord Microglia of SOD1-G93A ALS Mouse Model

**Figure 4:**
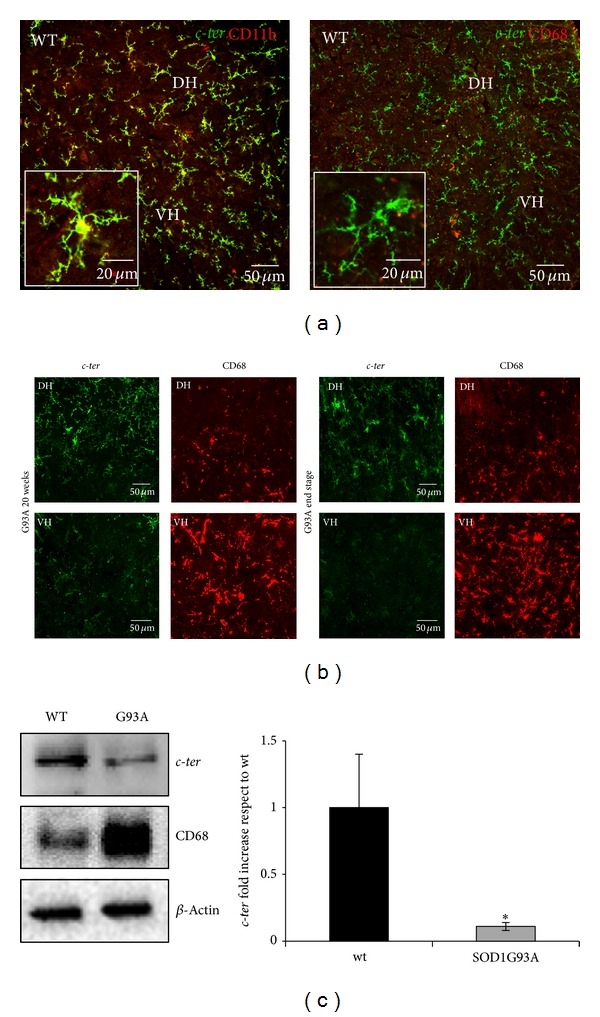
Temporal and regional pattern of P2Y_12_ expression in SOD1-G93A ALS spinal microglia. (a) Double immunofluorescence and confocal analysis on lumbar spinal cord sections (L3–L5) of wild-type (WT) mice was performed with* c-ter* antibody (green and yellow, merged and insets), CD11b (left panel, yellow, merged and inset), and CD68 (right panel, red, merged and inset), in both dorsal (DH) and ventral (VH) horns of spinal cord. (b) Double immunofluorescence and confocal analysis on SOD1-G93A lumbar spinal cord sections (L3–L5) at two different stages of ALS disease, that is, 20 weeks, and end stage, was performed with* c-ter* (green) and CD68 (red) antibodies. (c) Equal amount of total lumbar spinal cord lysates (L3–L5) from WT and SOD1-G93A (*n* = 4 for each group) were subjected to Western blotting and immunoreactions with* c-ter* and CD68 antibodies; anti-*β*-actin was used for protein normalization. Data represent means ± SEM. Statistical significance was calculated by Student's *t*-test, *P < 0.05.

In order to verify if P2Y_12_ recognized by* c-ter* antibody exclusively in microglia from rat brain slices could be used as specific microglia marker during neuroinflammation, we validated its use in a typical neuroinflammatory disease such as ALS and for the first time characterized the presence of P2Y_12_ receptor in SOD1-G93A mouse model (Figure 4). By immunofluorescence and confocal analysis on lumbar spinal cord sections (L3–L5) of wild-type (WT) mice, we first compared the immunoreactive signals obtained with* c-ter* and specific microglia markers CD11b (red, recognizing ramified microglia) and CD68 (red, recognizing roundish activated microglia). In WT mice,* c-ter *(green) is abundantly and strongly immunoreacting with the microglia population and colocalizing with the majority of CD11b-positive cells, in both dorsal (DH) and ventral (VH) horns of spinal cord (Figure 4(a), left panel, merged yellow signal). All CD11b-positive cells share immunoreactivity for* c-ter*, as proved by the absence of red CD11b signal. Conversely, not all* c-ter*-positive cells immunoreact also with CD11b antibody, as proved by the presence of some green* c-ter* signals. In addition, we never observe colocalization with the rare activated CD68-positive (red) microglia cells present in WT healthy tissue (Figure 4(a), right panel, merged and inset).

To test if* c-ter* antibody can further recognize microglia activation during the progression of ALS, we next examined SOD1-G93A spinal cord sections at two different stages of the disease, that is, 20 weeks, a phase when the disease accelerates, and end stage, that is, 23 weeks, when the animals reach the score of 1 accordingly to a behavioral score system [22]. At both stages, SOD1-G93A mice show a significant increase in CD68 immunostaining not only when compared to WT mice (Figure 4(a)) [22], but also in VH with respect to DH, and this is even more evident at end stage with respect to 20 weeks (red, Figure 4(b)). Remarkably, the immunoreactive signal of* c-ter* (green, Figure 4(b)) decreases during disease progression and the effect is pronounced especially in VH, where motor neuron loss, tissue damage, and microglia activation are known to be increased [2]. Particularly at 20 weeks,* c-ter*-expressing microglia are still ramified in DH, where few CD68-positive cells are present, but start to disappear in VH, where instead CD68-positive cells are increased. At end stage,* c-ter* staining decreases in DH with respect to 20 weeks and disappears almost completely in VH, concomitantly with a robust increase in CD68 staining. A parallel decrease in* c-ter* and increase of CD68 immunoreactivities is also confirmed by Western blot analysis performed on SOD1-G93A lumbar spinal cord homogenates at end stage (Figure 4(c)).

### 3.5. P2Y_12_ Expression Is Modulated during Neuroinflammation in MS Brain Lesions

**Figure 5:**
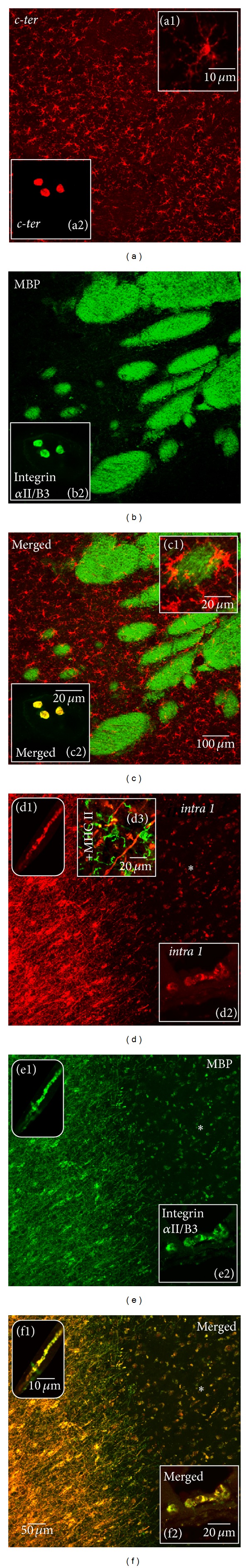
P2Y_12_ receptor in human cortex. Sections from human healthy and SPMS frontal cortex were analyzed by double immunofluorescence and confocal microscopy for the immunoreactive markers* c-ter* (red, panels (a), (c); insets a1, a2, c1; yellow merged, inset c2),* intra1* (red, panels (d), insets d1, d2, d3; yellow merged, panel f, insets f1, f2), MBP (green, panels (b), (c), (e), insets c1, e1; yellow merged, panel (f), inset f1), MHC II (green, inset d3), and integrin *α*II/*β*3 (green, insets b2, e2; yellow merged, insets c2, f2). The asterisks show decreased P2Y_12_ immunoreactivity in proximity to MS lesion.

As in rat organotypic cerebellar cultures (Figure 2(b)) and rat cerebellar, striatal, cortical, or mouse spinal cord tissue slices (Figures 3 and 4), a similar pattern of P2Y_12_ receptor expression is shown in human frontal cortex autoptic tissue by using* c-ter* antibody that highlights only microglia uniformly distributed throughout the entire healthy tissue (red), but not MBP-positive myelinated structures (green), as detected by the absence of overlapping immunofluorescent signals (Figures 5(a), 5(b), and 5(c), merged and insets a1, c1).

On the contrary,* intra1* (red, Figures 5(d), 5(e), and 5(f)),* intra2* [13], and* intra fl* (data not shown) antibodies recognize exclusively myelinated fibers in tissue from healthy (insets d1, e1, and f1) and progressive MS donors (Figures 5(d), 5(e), and 5(f)), as it is found with rat tissue (Figures 2 and 3). Moreover, we confirm a decrease of P2Y_12_ receptor signal in proximity to the demyelinating lesion, as detected by loss of MBP-positive fibers (see asterisks) [13]. No colocalization of signals is shown with MHC II-positive microglia (+ MHC II, inset d3, merged).

Importantly,* c-ter*,* intra1* (red, insets a-f2) and* intra2*, and* intra fl *(data not shown) antibodies all recognize the presence of P2Y_12_ receptor in integrin *α*II/*β*3-positive platelets (green) contained in the blood vessels of the analyzed tissues.

**Figure 6:**
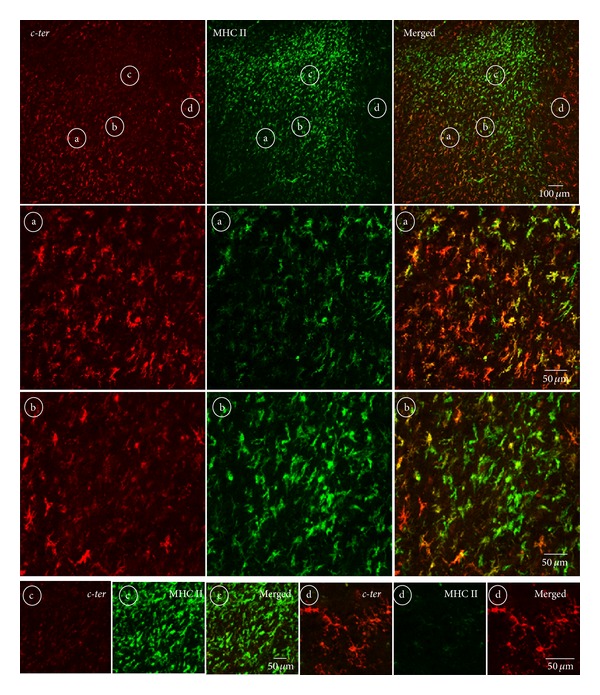
Regional distribution of P2Y_12_ in proximity to MS lesions. Sections from SPMS frontal cortex were analyzed by double immunofluorescence and confocal microscopy for* c-ter* (red) and MHC II (green) immunoreactivity. In proximity to the demyelinating active cortical lesion expressing augmented positivity for MHC II, microglia gradually lose immunoreactivity for* c-ter* antibody. Microglia express differential immunoreactivity in the four chosen areas which are found inside (circled b-c) and around (circled a–d) a lesion.

As observed in ALS mouse spinal cord where P2Y_12_ receptor detected by* c-ter* antibody is shown to temporally and regionally decrease in microglia as a function of increasing inflammatory damage (Figure 4), we notice that microglia gradually lose immunoreactivity for* c-ter* antibody (Figure 6) in proximity to the demyelinating active cortical lesions of MS expressing augmented positivity for MHC II [33–35]. Moreover, by comparing MHC II and* c-ter* signals, we recognize four areas (“a–d”) inside and around the lesion, where microglia express different amount of these proteins. In zone “a,” at the edge of the lesion, we observe a predominance of* c-ter* immunoreactivity (red) compared to that of MHC II (green), as depicted in the merged field by a major occurrence of red signal. In zone “b,” closer to the lesion, we notice a prevalence of active MHC II-positive green cells, but still the presence of few red and yellow signals. Finally, the extreme conditions are represented in zones “c” and “d,” apparently in the core or outside the lesion, respectively, where microglia express either almost exclusively MHC II protein (“c”) or P2Y_12_ receptor on ramified microglia (“d”).

## 4. Discussion

The interchange among different cell types of molecular cues that condition the cell specificity and the protein profile of each cell characterizes the morphological and functional heterogeneity in particular of microglia within various CNS regions [36], developmental stages [37, 38] and, even more, states of activation during pathological conditions [19]. In the case, for instance, of ALS, the release of signals from motor neurons apparently denotes one of the earliest phase of the disease, with microglia behaving as an M2 phenotype producing neuroprotective factors to repair motor neurons and preventing them against further injury [39]. As disease rises, motor neurons start releasing “alarm signals” that in turn convert microglia from beneficial M2 to cytotoxic M1 phenotype, with consequent release of proinflammatory cytokines. These often induce astrocytic dysfunction and further motor neuron degeneration [40]. In recent years, a dual functional phenotype of microglia has been identified also in MS. For instance, M1 markers are abundantly expressed in normal appearing white matter and throughout active demyelinating MS lesions by activated microglia and macrophages, although in human active MS lesions microglia show an intermediate activation status [41]. In addition, M2 microglia appear fundamental to guide oligodendrocyte remyelination in mice, and a switch from M1- to M2-dominant response occurs in microglia and peripherally derived macrophages when remyelination starts [42]. Only therapeutic procedures that both down-regulate the harmful responses and up-regulate the beneficial responses may hopefully slow pathological progression and provide meaningful hope for treatment. At the same time, the identification of clear markers involved in the M2/M1 microglia transition becomes mandatory for presymptomatic diagnosis, monitoring of disease progression, and efficacy of therapies.

Under this perspective, and consistently with previous findings establishing the role of purinergic receptors in the pathogenesis of both ALS and MS [14, 43], our present work serves this aim, by highlighting the gradual loss of P2Y_12_ immunoreactivity as an early marker of neuroinflammation and microglia metamorphosis. We have indeed demonstrated here that P2Y_12_ receptor protein identified in primary cultures of both microglia (Figure 2(a)) [18, 44] and oligodendrocytes (Figure 2(b)) [15] by different but P2Y_12_-selective antibodies can be instead recognized in branched microglia exclusively by the use of* c-ter* antibody (Figures 3–6). This occurs only when the integrity and cytoarchitecture of the tissue is typically preserved in the presence of the least experimental manipulations, that is, in organotypic cultures (Figure 2(c)) and tissues slices for instance from rat striatum and cerebellum (Figure 3), mouse spinal cord (Figure 4), and human cerebral cortex (Figures 5 and 6). A similar difference in primary cultured cell versus tissue distribution of a protein was previously demonstrated with large-conductance calcium-activated potassium channel expression, in vascular endothelium [45]. A first implication emerging from these results is that a reliable evidence about selective P2Y_12_ expression in cells of healthy or neuroinflammatory states is genuine only when cell connectivity and tissue architecture are fully preserved. We have indeed shown this, by proving that the antibodies used for P2Y_12_, recognizing either the C-terminus or the second intracellular loop of the receptor (Figure 1 and Table 1) and immunolabelling, respectively, microglia or myelinated fibers in the CNS, are all still able to immunoreact for instance with platelets (Figure 5), where the receptor was originally described to be present and to have a role in the processes of activation, aggregation [46–49], primary hemostasis, and arterial thrombosis [50–55]. A possible explanation for the microglia versus oligodendrocyte selectivity of the P2Y_12_ antibodies might be that Gi-coupling, and/or quaternary structure, post-transcriptional modifications, and subcellular localization of P2Y_12_ that remain strictly preserved in platelets, are instead divergent in microglia with respect to oligodendrocytes/myelinated fibers. In this case, a cell-specific network of P2Y_12_ oligomeric interactions and/or a distinct subcellular partitioning might simply mask the recognition sites of the different antibodies on P2Y_12_ protein in different cell types. To support this hypothesis, we know that in platelets P2Y_12_ indeed resides in subcellular lipid raft structures and its partitioning out from rafts causes for instance inactivation [56] and that also the presence of another purinergic receptor, the ionotropic P2X_3_, in lipid rafts has cell-specific properties shared in cerebellar granule neurons and total brain tissue but not in neuroblastoma cells and dorsal root ganglia [57] and that the specific antagonist clopidogrel inhibits P2Y_12_ by breaking down the homoligomeric complex to single monomers [58] and finally, that hetero-oligomerization of P2Y_12_ is demonstrated with P2Y_1_, P2Y_2_, P2Y_13_, and with adenosine A_1_, A_2A_ receptors in different cellular backgrounds [59]. All these features might very well explain also why it has not been found colocalization between P2Y_12_ and GFAP-positive astrocytes in rat brain cortex and nucleus accumbens, despite the abundant presence of P2Y_12_ mRNA [11] and, moreover, why P2Y_12_ is specifically observed in brain and spinal cord resident microglia but is not observed, for example, in peripheral macrophages in spleen [18, 20].

**Figure 7:**
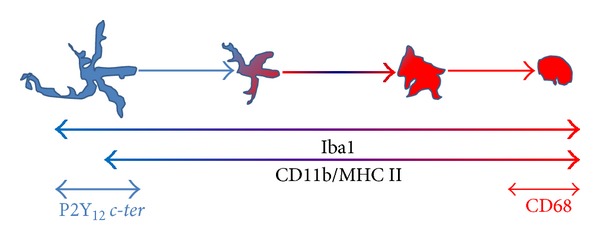
Draw of microglial marker expression as a function of activation. Branched microglia are represented in blue and activated microglia in red. Iba1 [22], CD11b, and MHC II are mostly expressed in microglia throughout the different morphological states and their expression increases during activation (light blue to red color). P2Y_12_ *c-ter* (light blue) and CD68 (red) are expressed, respectively, in branched or roundish/activated microglia.

A second implication that emerges from our results is that the morphological metamorphosis that microglia undergo under neuroinflammatory conditions as those triggered during ALS and MS, can be remarkably highlighted by the progressive reduction of P2Y_12_ immunostaining obtained with* c-ter* antibody that reacts, also in this case, exclusively with multibranched microglia still present in the tissue (Figures 4 and 6). This closely reflects the expression of P2Y_12_ that is robust in the resting/surveillant branched state but dramatically decreased after morphological transition and activation of microglia [18]. Our observations are also in line with the central role played by P2Y_12_ in branched microglia membrane ruffling and inspection of the environment [60]. On the other hand, they depict a morphological/functional state of microglia that only partially overlaps with CD11b and MHC II immunoreactivities, which are furthermore known to increase during activation but instead highly contrasts with CD68 immunostaining that is totally absent in ramified microglia [19]. In parallel to our results, these last antibodies actually accentuate the progressive transition of microglia from a lesser ramified shape to a significantly more activated amoeboid phenotype (Figures 4, 6, and 7), thus suggesting the dual use of* c-ter* and CD68 antibodies as markers, respectively, for branched resting/surveillant versus roundish/activated microglia. A reduction of* c-ter*-P2Y_12_ immunostaining that is concomitant to an increase, for instance, of MHC II/CD11b/CD68 immunoreactivity, could thus become a feasible approach to detect an increasing neuroinflammatory condition. Indeed, P2Y_12_ is considered an essential component and primary site at which nucleotides such as ADP act to promote directional microglia movement or chemotaxis at early stages of CNS injury [17]. In particular, microglia from mice lacking P2Y_12_ exhibit normal baseline motility but are unable to polarize and to elicit directional branch extension and migration toward nucleotides* in vitro*, or sites of cortical damage* in vivo* [18]. These notions are consistent with our results that fail to describe* c-ter*-P2Y_12_ immunoreactivity on roundish phagocytizing, or polarized migratory microglia. However, we still do not know if reduction/absence of P2Y_12_ *c-ter*-immunolabelling on activated microglia might be a cause or a consequence of morphological/functional transition or might simply reflect a cell-selective hindrance and lack of access to the immunogenic sites by the antibody. Further work will clarify this issue. Anyhow, we can assert that the distinctive recognition of multibranched microglia renders the* c-ter* antibody a novel and useful tool to discriminate among microglia morphological states, thus making P2Y_12_ receptor a selective and early marker for the ramified phase.

In parallel to this, we have also proven that all the several antibodies raised against the second intracellular loop of P2Y_12_ (*intra1*,* intra2*, and* intra fl*) can likely be employed as markers for the presence of MS lesions. Although with different intensities, they not only recognize the receptor specifically on myelinated fibers of organotypic cultures (Figure 2(c)), tissues slices from rat striatum or cerebellum (Figure 3) and human cerebral cortex, but also furthermore highlight the reduction of P2Y_12_ signal that occurs for instance in MS tissue (Figure 5) in correlation to the extent of demyelination found in all types of grey matter cortical plaques (I–III) and subcortical white matter [13].

In brief, we have shown here that the presence of P2Y_12_ receptor can be simultaneously identified by the C-terminus and the second intracellular loop antibodies. When this occurs, a condition of intact myelinated fibers and branched/surveillant microglia is represented at once that perhaps signifies a “healthy” state of the analyzed tissue. Any deviance from this picture likely characterizes a neuroinflammatory condition. For instance in MS, a decrease in the second intracellular loop immunoreactions accompanied by an increase of C-terminus immunoreactivity will possibly depict the loss of myelin and replacement by ramified microglia that often occur in an inactive plaque. On the contrary, a decrease in C-terminus immunolabelling in the abundant presence of second intracellular loop-positive myelinated fibers would indicate an early active plaque where M1/M2 microglia reactivity starts to take place.

## 5. Conclusion

By comparative analysis of all the available P2Y_12_-immunoreactive antibodies recognizing the C-terminus or the second intracellular loop of the receptor, we have established here that, under experimental conditions of well-preserved cytoarchitecture and tissue integrity, P2Y_12_ receptor expressed by both ramified microglia or oligodendrocytes/myelinated fibers might serve a dual function as specific marker, respectively, of branched/surveillant microglia as well as demyelinating lesions. We believe that P2Y_12_ identification and modulation might potentially acquire an important predictive value under neuroinflammatory conditions, as those found in ALS and MS. P2Y_12_ is likely to deserve a key role in the verge of a neuroinflammatory breakdown.

## Figures and Tables

**Table 1:** Primary antibodies employed in the study.

Antigen	Clone	Epitope (aa)	Target	Dilution	Source
MBP	2	119–131	Mature oligodendrocytes/myelin	1 : 100	Chemicon
SMI94	SMI-94	70–89	Mature oligodendrocytes/myelin	1 : 1000	Covance
NG2 chondroitin sulfate proteoglycan	132.39	∗	Oligodendrocyte precursor cells	1 : 200	Chemicon
CD11b rat	OX-42	∗	Microglia/macrophages	1 : 200	AbD Serotec
CD11b mouse	5C6	∗	Microglia/macrophages	1 : 200	AbD Serotec
CD68	FA-11	∗	Macrophages/monocytes	1 : 200	AbD Serotec
HLA-DP, DQ, DR (MHC II)	CR3/43	∗	Microglia/macrophages	1 : 100	Dako
Integrin *α*II/*β*3	(A2A9/6)	Full length	Platelets	1 : 100	Santa Cruz
NeuN	A60	∗	Neurons	1 : 200	Millipore
GFAP	G-A-5	∗	Astrocytes	1 : 400	Sigma
P2Y_12_ receptor (*intra1*)	Polyclonal	125–142	P2Y_12_ receptor	1 : 200	Sigma
P2Y_12_ receptor (*intra2*)	Polyclonal	125–142	P2Y_12_ receptor	1 : 200–300	Alomone
P2Y_12_ receptor-ATTO-594 (*intra fl*)	Polyclonal	125–142	P2Y_12_ receptor	1 : 50	Alomone
P2Y_12_ receptor mouse/rat (*c-ter*)	Polyclonal	C-terminus [18]	P2Y_12_ receptor	1 : 200	Anaspec
P2Y_12_ receptor human (*c-ter*)	Polyclonal	324–342	P2Y_12_ receptor	1 : 200	Anaspec

CD11b: cluster of differentiation 11b; CD68: cluster of differentiation 68; fl: fluorescent at 594 nm; GFAP: glial fibrillary acidic protein; HLA: human leukocyte antigen; MBP: myelin basic protein; MHC: major histocompatibility complex; NeuN: neuronal nuclei; NG2: neural/glial antigen 2.

∗Not specified in the data sheet.

## Abbreviations

ALS: Amyotrophic lateral sclerosis
CD11b: Cluster of differentiation 11b
CD68: Cluster of differentiation 68
DH: Dorsal horn
GFAP: Glial fibrillary acidic protein
MHC II: Major histocompatibility complex II
MBP: Myelin basic protein
MS: Multiple sclerosis
NeuN: Neuronal nuclei
NG2: Neural/glial antigen 2
NDS: Normal donkey serum
SPMS: Secondary progressive multiple sclerosis
PBS: Phosphate buffer saline
VH: Ventral horn.
